# Killing three birds with one BPI: Bactericidal, opsonic, and anti-inflammatory functions

**DOI:** 10.1016/j.jtauto.2021.100105

**Published:** 2021-05-28

**Authors:** Jomkuan Theprungsirikul, Sladjana Skopelja-Gardner, William F.C. Rigby

**Affiliations:** a Department of Microbiology and Immunology, Geisel School of Medicine at Dartmouth, Lebanon, NH, United States; b Division of Rheumatology, Department of Medicine, Geisel School of Medicine at Dartmouth, Lebanon, NH, United States

**Keywords:** Bactericidal/permeability-increasing protein (BPI), Gram-negative bacteria, Opsonization, Antimicrobial peptides, Anti-inflammation, Autoantibody

## Abstract

Bactericidal/permeability-increasing protein (BPI) is an anti-microbial protein predominantly expressed in azurophilic granules of neutrophils. BPI has been shown to mediate cytocidal and opsonic activity against Gram-negative bacteria, while also blunting inflammatory activity of lipopolysaccharide (LPS). Despite awareness of these functions *in vitro,* the magnitude of the contribution of BPI to innate immunity remains unclear, and the nature of the functional role of BPI *in vivo* has been submitted to limited investigation. Understanding this role takes on particular interest with the recognition that autoimmunity to BPI is tightly linked to a specific infectious trigger like *Pseudomonas aeruginosa* in chronic lung infection. This has led to the notion that anti-BPI autoantibodies compromise the activity of BPI in innate immunity against *P. aeruginosa*, which is primarily mediated by neutrophils. In this review, we explore the three main mechanisms in bactericidal, opsonic, and anti-inflammatory of BPI. We address the etiology and the effects of BPI autoreactivity on BPI function. We explore BPI polymorphism and its link to multiple diseases. We summarize BPI therapeutic potential in both animal models and human studies, as well as offer therapeutic approaches to designing a sustainable and promising BPI molecule.

## Highlights

• Bactericidal, opsonic, and anti-inflammatory functions of BPI were summarized.
• Etiology and effects of BPI autoreactivity on BPI function were explored and addressed.
• BPI polymorphism and its link to multiple diseases were explored and discussed.
• BPI therapeutic potential in both animal models and human studies was summarized.
• Therapeutic approaches to designing a sustainable and promising BPI molecule were introduced.

## 1 Introduction

Patients with cystic fibrosis (CF), bronchiectasis, and chronic obstructive pulmonary disease (COPD) characterized by persistent airway infection by *Pseudomonas aeruginosa* exhibit increased morbidity and mortality [[1], [2], [3], [4], [5], [6], [7]]. Moreover, impaired lung function in these chronic lung diseases is independently associated with the production of autoantibodies against bactericidal/permeability-increasing protein (BPI) [[7], [8], [9], [10]]. BPI is a protein of ~55 kDa and is stored in primary azurophilic granules of neutrophils [11,12]. Upon neutrophil encounter of Gram-negative bacteria, BPI is released to mediate bactericidal effects, phagocytosis, and uptake of bacteria by neutrophils and dendritic cells (DCs), while neutralizing the inflammatory activity of lipopolysaccharide (LPS) [11,[13], [14], [15], [16], [17], [18], [19]]. Given the strong association between *P. aeruginosa* chronic infection and autoreactivity to BPI [8,[20], [21], [22]], it is possible that autoantibodies to BPI enable bacteria to evade the immune response, contributing to worsening disease state in these patients. In this review article, we summarize and explore the functional biology of BPI in innate immunity and the possible modulation of this pathway by adaptive immune responses.

## 2 Structural and functional characteristics of BPI

**Fig. 1:**
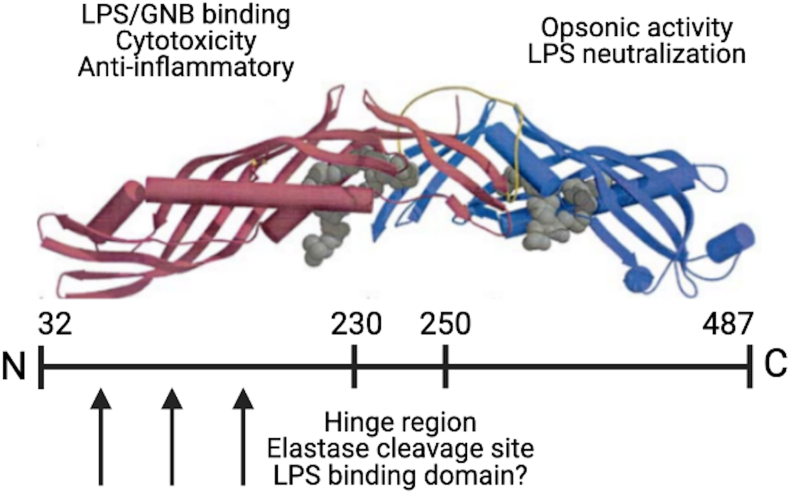
**Structural and functional characteristics of BPI.** BPI (amino acids 32–487 after signal sequence cleavage) mediates at least three innate immune activities. Separate functional activities exist in the N-terminus and C-terminus. LPS binding enables direct cytocidal activity against gram-negative bacteria (GNB) and blocks LPS-induced TNF. The N-terminus (aa 32–230) has three LPS binding sites (black arrows). The C-terminus (aa 250–487) opsonizes *P. aeruginosa* bound to the N-terminus and is necessary for clearance of *P. aeruginosa in vivo*. The C-terminus and hinge (aa 231–249) region exhibits LPS binding activity *in vitro* suggesting their possible contribution to *P. aeruginosa* clearance. Structure of BPI shown here was reported previously [24].

BPI, first purified and characterized by Weiss et al., in 1978 [11,12], is a cationic antimicrobial protein, part of the BPI-fold containing (BPIF) superfamily [23,24]. Connected by a central beta pleated-sheet, the protein exhibits N- and C-terminal barrel-shaped domains that are structurally similar despite a low level of amino acid identity [23,24] (Fig. 1). A separate branch of this superfamily is the Palate Lung and Nasal epithelium Clone (PLUNC) proteins, whose expression is chiefly limited to the airway epithelial cells in vertebrates [25]. BPI is detectable at the promyelocyte stage of myeloid development [11] and subsequently found in azurophilic granules of neutrophils and to a lesser extent in eosinophils, dermal fibroblasts, macrophages, and certain mucosal epithelial cells [1,12,[26], [27], [28], [29]], although dermal fibroblast and epithelial cells require stimulation for BPI expression [27,29].

The N-terminal domain of BPI binds lipopolysaccharide (LPS) with nanomolar affinity [[30], [31], [32]]. Neutrophil degranulation releases BPI wherein its N-terminal domain mediates binding to the negatively charged lipid A moiety of LPS expressed on the bacterial outer envelope [30,33]. This interaction destabilizes the integrity of the bacterial membrane, leading to bacterial lysis and cell death in solution [8,12,13,30,[33], [34], [35]]. The C-terminal portion of BPI has been implicated in LPS binding and opsonization but these activities are less well understood [36,37]. BPI additionally mediates intracellular killing of Gram-negative bacteria following phagocytosis via fusion with phagolysosome containing secreted BPI [38]. The presence of BPI has also been reported in neutrophil extracellular traps, providing yet another route for bacterial killing and clearance [39,40].

The N-terminal domain of human BPI (amino acids 1–230) is linked to the C-terminal domain (amino acids 250–456) of BPI by a proline-rich hinge region (amino acids 230–250) that also contains an elastase cleavage site (amino acids 240–245) [23]. As stated above, both domains are of similar size, secondary structure, and topology which give rise to its boomerang shape. Finally, recent work suggests that BPI also binds Gram-positive *Staphylococcus aureus* lipopeptides with nanomolar affinity [32]. Binding of these lipopeptides is blocked by LPS, suggesting the N-terminal BPI domain also interacts with Gram-positive cocci [32]. Moreover, BPI was shown to enhance the immune response (elevated levels of TNFα, IL-6 and IL-8) toward Gram-positive ligands in peripheral blood mononuclear cells (PBMCs) when synthetic bacterial lipopeptides, lipoteichoic acid (major cell wall component of Gram-positive bacteria), and lysates of Gram-positive bacteria were used [32]. Interestingly, BPI was not shown to have a direct bactericidal effect on Gram-positive bacteria in previous studies [12,41].

## 3 Direct cytocidal activity of BPI

It has been shown that neutrophils derived from newborn umbilical cord blood express less BPI than those from adults [42,43]. Moreover, newborn neutrophils are defective in their phagocytic and bactericidal activity against *S. aureus* and *Escherichia coli* [44]. Acid extracts of newborn neutrophils also exhibit decreased antibacterial activity against serum-resistant *E. coli* [43], rendering them less effective in these infections. Chronic granulomatous disease (CGD) neutrophils exhibit defective phagocyte NADPH oxidase and hence, reduced antimicrobial hydrogen peroxide production [45]. Despite this defect against Gram-positive cocci like *S. aureus*, neutrophils from CGD patients are capable of killing *E. coli* [38], suggesting that BPI mediates bacterial clearance independent of reactive oxygen species (ROS) [38,45]. Moreover, purified antibodies to BPI from human serum has been shown to inhibit BPI (from neutrophil extracts) from killing *E. coli* [46], further validating the independent cytocidal activity of BPI.

**Fig. 2:**
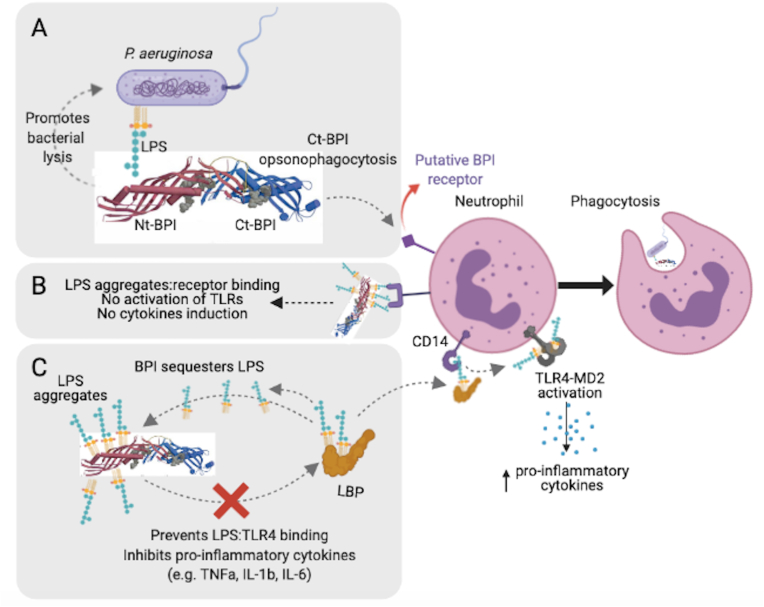
**Proposed model of BPI functions in bactericidal, anti-inflammatory, and opsonophagocytosis of *Pseudomonas aeruginosa*. (A)** The cationic N-terminal portion of BPI binds to the negatively-charged LPS contained in the outer membrane of *P. aeruginosa,* and promotes bacterial lysis by destabilizing integrity of bacterial membrane. By binding to LPS, BPI also acts to inhibit pro-inflammatory cytokines released by the host through LPS neutralization. The C-terminal portion of BPI opsonizes *P. aeruginosa* through a putative BPI receptor on neutrophil, resulting in opsonophagocytosis of the bacteria. Presence of BPI is critical due to its anti-inflammatory and phagocytosis induction properties. **(B)** LPS aggregates bind to a receptor on neutrophils but do not activate TLR and therefore, no pro-inflammatory cytokines are released. **(C)** LPS-binding protein (LBP) catalyzes and disperses LPS aggregates and delivers the monomers to CD14/TLR-4 receptor complexes, triggering the release of pro-inflammatory cytokines. Due to its high affinity for LPS, BPI increases the size of LPS aggregates, thereby sequestering LPS from interacting with LBP and blunting inflammatory activities of monocytes by CD14-independent and dependent mechanisms. Structure of BPI shown here was reported previously [24].

Overall, most evidence points to the N-terminus as solely responsible for the bactericidal activity of BPI. When the cationic BPI N-terminus binds to the negatively charged LPS phosphate groups (Fig. 2), this action displaces divalent cations which disturbs the arrangement of LPS molecules and the bacterial membrane potential, causing membrane rupture [33]. This BPI-mediated outer membrane damage halts bacterial growth and allows cellular entry of other synergistic anti-microbial peptides, such as cathelicidins and defensins [13]. Additionally, BPI also acts in synergy with the complement system as BPI bactericidal activity toward *E. coli* is inhibited by C7-depleted serum but accelerated by normal serum [47,48]. Following outer membrane damage, subsequent phospholipid hydrolysis and disruption of the inner bacterial membrane ultimately lead to bacterial killing [49]. That BPI binding to LPS is essential for bacterial killing is supported by a study showing Gram-negative bacteria *Proteus mirabilis* are less susceptible to BPI killing, presumably due to steric hindrance of BPI access to the lipid A in the long polysaccharide chains with tightly packed LPS seen in this organism [50]. The targeting of LPS gives rise to bacterial mutations, such as fatty acid additions and hydroxylation or acetylation of the O-antigen, in an attempt to modify their LPS composition and structure to avoid direct target and killing by bactericidal proteins [51]. Interestingly, despite the ability of BPI to bind to lipopeptides/lipoproteins associated with Gram-positive cocci, direct cytocidal activity is not evident unless lipoteichoic acid (LTA), another major component of the cell wall of Gram-positive bacteria, is present, as LTA was shown to be an additional ligand of BPI in Gram-positive bacteria [32]. Moreover, there has been evidence showing that L-forms (i.e. lacking cell wall) of the Gram-positive bacteria *Staphylococcus aureus* and *Streptococcus pyogenes* are susceptible to BPI inhibition and killing due to lack of protection of the cytoplasmic membrane [52].

## 4 Opsonophagocytic activity of BPI

While the bactericidal activity of BPI has been the main focus of the *in vitro* functional studies, the opsonophagocytic function of BPI has not been well understood. Though the N-terminal domain mediates the BPI:LPS binding, the structurally similar carboxy-terminal domain is thought to mediate bacterial opsonization [8,36,37]. The C-terminal BPI fragment was also shown to inhibit inflammation triggered by endotoxin, this activity required 5–10 fold higher molar protein concentrations than the N-terminal domain [31]. Gram negative bacteria, *E. coli* K1/r, pre-incubated with native human BPI are ingested by neutrophils and monocytes [36]. This activity is missing with the recombinant N-terminal domain (rBPI_21_), suggesting the C-terminus promotes bacterial phagocytosis, possibly via direct binding (Fig. 2). This model was supported by the observation that the uptake of Gram-negative bacteria by DCs is promoted by the C-terminal domain [8]. However, addition of the recombinant BPI (full length) to serum promotes phagocytosis of *E. coli* but not *S. aureus* by promoting complement activation (deposition of C3b/iC3b fragments) on the bacterial surface, possibly indicating indirect effects of BPI on phagocytosis [53]. The importance of the opsonophagocytic role of BPI has recently been highlighted by the *in vivo* studies using BPI-deficient mice. The absence of BPI impaired neutrophil phagocytosis and clearance of *P. aeruginosa* in acute infection. The ability of BPI-deficient mice to clear *P. aeruginosa* was corrected with the administration of neutrophil-purified human BPI [129]. Intracellular uptake of exogenous BPI and *P. aeruginosa* complex was observed, reinforcing the notion that BPI mediates phagocytosis *in vivo* [129]. Recent study has shown that CD18, a β2 integrin expression facilitates the uptake of both motile and nonmotile *P. aeruginosa* strains by phagocytes [54]. BPI-enhanced phagocytosis and *P. aeruginosa* clearance were inhibited by CD18 blockade *in vivo.* These data further supports evidence of phagocytosis rather than bactericidal activity of BPI [129]. Therefore, BPI not only facilitates leukocyte clearance of Gram-negative bacteria, but it also promotes antigen uptake and presentation, which can serve as a necessary link between innate anti-bacterial defenses and induction of adaptive immune responses [55].

## 5 Anti-inflammatory activity of BPI

In addition to facilitating the clearance of Gram-negative bacteria, BPI has been reported to exhibit anti-inflammatory effects by regulating LPS-triggered cytokine responses (Fig. 2). BPI belongs to the family of lipid-transfer proteins mentioned earlier including LPS binding protein (LBP) that is present in normal serum [56,57]. However, unlike LBP, which facilitates pro-inflammatory activation of monocytes by LPS, BPI binding to LPS blunts its ability to trigger endotoxin activation [58]. LBP catalyzes and disperses LPS aggregates and delivers the monomers to CD14/TLR-4 receptor complexes, triggering the release of pro-inflammatory cytokines [58]. Due to its high affinity for LPS, BPI increases the size of LPS aggregates, thereby sequestering LPS from interacting with LBP and blunting inflammatory activities (i.e. pro-inflammatory cytokine production and release) of monocytes by CD14-independent and dependent mechanisms [37,[58], [59], [60]] (Fig. 2). This anti-inflammatory effect is seen with the N-terminal domain [31,61] and in one study the C-terminal domain alone [31]. Interestingly, despite the ability of BPI to bind to lipopeptides and lipoproteins on *S. aureus,* no inhibition of cytokine release is seen, suggesting specificity of BPI effects on LPS-mediated activation and cytokine release [32]. In fact, there was a dose-dependent increase in TNFa, IL-6, and IL-8 secretion with increasing concentration of BPI in the presence of lipopeptides and lipoproteins, suggesting that BPI enhances the immune response toward Gram-positive ligands [32].

Recent *in vivo* studies of acute *P. aeruginosa* infection have demonstrated enhanced neutrophil recruitment and inflammatory cytokine production in the absence of BPI [129]. Administration of exogenous human BPI reduced cellular inflammation and cytokine production (TNF, IL-6, IL-1b) at the site of infection [129]. Therefore, besides mediating specific and direct killing of Gram-negative bacteria and facilitating bacterial opsonization, BPI also mediates endotoxin neutralization via mechanisms that simultaneously work to eradicate bacterial infection and to dampen excessive inflammation.

## 6 Autoantibodies to BPI and their impact on BPI-dependent immunity

### 6.1 Etiology

Mysteriously, BPI and other contents of the azurophilic granules have been recognized as frequent targets of humoral autoimmunity [62]. Anti-neutrophil cytoplasmic autoantibodies (ANCA) are found in primary vasculitic syndromes, granulomatosis and polyangiitis (GPA) and microscopic polyangiitis (MPA) exhibit specificity for azurophilic granular proteins proteinase 3 (PR3) and myeloperoxidase (MPO) [[63], [64], [65], [66]]. These relationships prompted the discovery of BPI-ANCA in a subset of vasculitis patients in the early 1990s [67,68]. Despite their shared origin in neutrophil granules, the etiology and the specificity of these responses are not understood [69]. Defects in the progression of apoptosis or in the removal of apoptotic cells [[69], [70], [71]] have been proposed for the production of ANCA *in vivo*. In this regard, the presence of these autoantigens on neutrophil extracellular traps (NETs) is proposed to lead to the breaking of tolerance to self-protein [72]. ANCA targeting of different NET-associated bactericidal proteins rarely track together despite the fact that those proteins are localized to the same neutrophil azurophilic granules [73,74], suggesting the disease- or infection-specific nature of ANCA. Autoantibodies to BPI have a remarkable restriction to *P. aeruginosa* infection, and presence of BPI antibodies is associated with worse disease outcome [6,10,14].

**Fig. 3:**
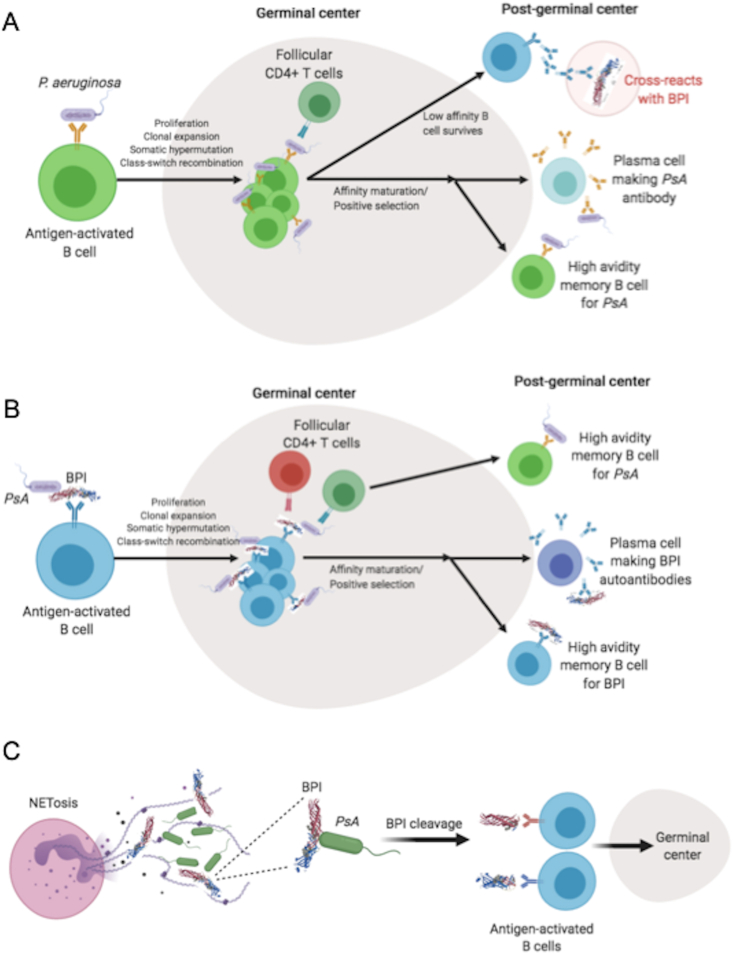
**Proposed models of the etiology of anti-BPI autoantibodies. (A)***Molecular mimicry*: Antigen-activated B cell captures *P. aeruginosa*, undergoes proliferation, clonal expansion, somatic hypermutation, and class-switching in the germinal center before affinity maturation into high avidity plasma cell and memory B cell targeting *P. aeruginosa*, which can cross-react with BPI antigen. **(B)** BPI:*P. aeruginosa* complex enhances uptake of BPI into the germinal center. Antigen-activated B cell captures the complex, undergoes proliferation, clonal expansion, somatic hypermutation, and class-switching in the germinal center. The BPI-*P. aeruginosa* antigens are presented to the T cells, going through class switching and affinity maturation to make high avidity memory B cells and plasma cells targeting either BPI and *P. aeruginosa* antigens. **(C)***Generation of cryptic epitopes* of cleaved BPI through interaction of *P. aeruginosa* elastase and BPI elastase-sensitive region (amino acids 240–245). Newly generated cryptic BPI epitopes then get picked up by antigen-activated B cells before going through proliferation in the germinal center. Structure of BPI shown was reported previously [24]. *PsA* represents *P. aeruginosa*.

Anti-BPI autoantibodies have been peculiar for a number of reasons, most notably their strong linkage with patients suffering from various diseases, particularly cystic fibrosis (CF) [10,75,76] and bronchiectasis [6,67], but also including inflammatory bowel diseases (IBD) [63,77,78], vasculitis [63,67,68,79], reactive arthritis [80], necrotizing and crescentic glomerulonephritis [81], and primary sclerosing cholangitis [82,83]. The etiopathogenesis of BPI autoantibodies is unknown. Three possible models for breaking of immune tolerance to BPI have been proposed: i) molecular mimicry, ii) cross-activation of immune response to BPI:bacterial complex, and iii) immune response to BPI cryptic epitope generated from interaction with bacteria [6,10] (Fig. 3A–C).

i) The relationship of autoimmunity to specific infection remains obscure except for *P. aeruginosa* particularly in the lung. Our recent studies have indicated molecular mimicry as the less likely mechanism. In a cohort of bacteremic patients, we showed that BPI autoantibodies were present in patients with Gram-positive as well as Gram-negative sepsis [84]. Anti-BPI antibodies in bacteremic patients (acute infections) were of low-avidity [84], compared to those in CF or bronchiectasis (chronic infections) patients [6,10], suggesting the breaking of tolerance to BPI arises through affinity maturation rather than cross-reactivity to *P. aeruginosa* [10] (Fig. 3B and C). Thus, high avidity anti-BPI antibodies are restricted to patients with chronic lung infection by *P. aeruginosa.* In contrast, IBD patient sera frequently exhibits anti-BPI reactivity, these autoantibodies are of low-avidity in contrast to that seen in the lung infection by *P. aeruginosa* (unpublished observation).
ii) Apart from the lung infection and its relationship to ANCA, colonic mucosal levels of BPI are increased in IBD patients [85] and are associated with anti-BPI antibodies in ulcerative colitis patients [77]. However, while higher BPI protein levels are reported in serum of bacteremia patients, there was no correlation between serum BPI protein levels and anti-BPI IgG responses [84]. This evidence suggests a requirement of both presence of BPI and chronic infection/inflammation conditions for the BPI autoantibodies to be generated.
iii) There has been evidence suggesting that exposure of BPI cryptic epitope generated from *P. aeruginosa* interactions (i.e. *P. aeruginosa* elastase) [39] leads to generation of autoantibodies to BPI. We showed that presence of cleaved BPI protein in bronchoalveolar lavage (BAL) samples of CF patients is strongly associated with IgA antibodies to *P. aeruginosa* and BPI [10]. This evidence is consistent with a model by which cleaved BPI antigen formed in the BAL arises in the presence of chronic airway infection by *P. aeruginosa*, and contributes to the breaking of tolerance to BPI in the lungs. This model has been described in Fig. 3C. Additionally, the presence of *P. aeruginosa* infection in the airways could lead to increased neutrophil recruitment to the infection site. Since neutrophil elastase is also contained within neutrophil azurophilic granules alongside BPI [86,87], the concomitant release of both BPI and neutrophil elastase from the activated neutrophils could play a role in cleaving BPI at its elastase sensitive site [23], exposing its cryptic epitope.

### 6.2 Effects on BPI function

**Fig. 4:**
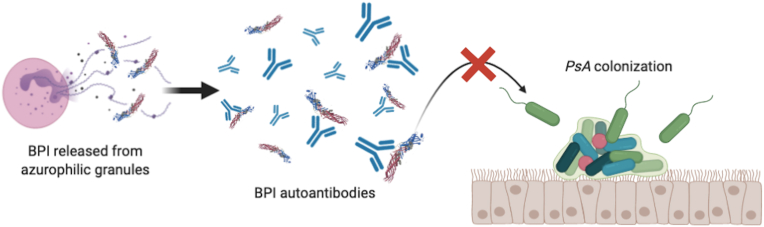
**Presence of BPI autoantibodies compromises the bactericidal effect of BPI.** Anti-BPI autoantibodies neutralize the ability of BPI to bind to LPS and kill *P. aeruginosa*, allowing the persistence of *P. aeruginosa* infection in cystic fibrosis, bronchiectasis, and chronic obstructive pulmonary disease. Structure of BPI shown here was reported previously [24]. *PsA* represents *P. aeruginosa*.

In the presence of BPI autoantibodies, studies in both European and United States CF cohorts have proposed that the innate immune system fails to combat airway *P. aeruginosa* infection [19,20,39,88]. Anti-BPI autoantibodies strongly associate with severity of disease, including poor lung function in CF, bronchiectasis, and COPD [6,7,10]. The strong association between high-avidity anti-BPI autoantibodies with chronic *P. aeruginosa* airway infection [8,[20], [21], [22]], suggests that the efficiency of neutrophils to clear Gram-negative bacteria may be compromised by the autoantibodies to BPI (Fig. 4).

Several *in vitro* studies have attempted to delineate the functional effects of BPI autoantibodies. Goat anti-BPI antibodies neutralize the antibacterial activity of BPI against *E. coli* [89]. Anti-BPI autoantibodies purified from CF or IBD sera prevent BPI-mediated phagocytosis and inhibit neutrophil-mediated killing of Gram-negative bacteria [19,78,[90], [91], [92]]. Anti-BPI antibodies against both the N- and the C-terminal domains isolated from IBD patient sera were able to inhibit bactericidal activity of BPI, and were associated with a more aggressive disease in IBD [78]. For CF, the majority (72%) of anti-BPI antibodies were specific to the C-terminus [19]. While these *in vitro* studies and strong clinical correlations suggest autoantibody-mediated inhibition of BPI function, they do not establish clear functional consequences of autoreactivity to BPI*,* let alone the phenotype of anti-BPI antibodies in neutralizing BPI function and facilitating bacterial persistence *in vivo.* Having demonstrated functional non-redundancy of BPI in combating *P. aeruginosa* infection *in vivo* [129], we can now interrogate the role for BPI autoantibodies in both acute and chronic murine infection models. These approaches will also allow us to investigate the etiology of anti-BPI autoantibodies *in vivo*.

## 7 BPI gene polymorphisms and their link to disease

IBD such as Crohn's disease (CD) and ulcerative colitis (UC) are chronic relapsing inflammatory disorders of the intestine in which the underlying pathophysiology is unknown. Other than in neutrophils, BPI is also produced on the apical and apicobasal surfaces of the human gastrointestinal epithelium [91]. Interestingly, BPI protein concentrations are elevated in gut tissue of UC patients [85,93,94], and BPI-ANCA are elevated with increasing severity of clinical status in IBD [77]. Previously, a gene variant polymorphism of BPI (Lys216Glu) was described in a case control study of sepsis patients [95]. Subsequently the Lys216Glu polymorphism was shown to be associated with both CD and UC [[96], [97], [98]]. It has been speculated that any impairment in the functions of BPI protein through BPI gene polymorphism may alter pathogen recognition by the innate immune system, eventually giving rise to undesirable outcomes in IBD [97], potentially due to gut dysbiosis.

Additional SNPs in the same superfamily of proteins have been associated with increased infection. Bactericidal/permeability-increasing protein fold–containing family member A1 (BPIFA1), formerly known as SPLUNC1, is one of the most abundant proteins produced by epithelial cells of the upper and proximal lower respiratory tract [99,100]. BPIFA1 single-nucleotide polymorphisms (G allele of rs1078761) is reported to be associated with decreased expression of BPIFA1 and with reduced lung function and more severe disease in CF [101,102]. BPI mutation PstI (T→C) polymorphism in intron 5 was associated with an increased risk of developing COPD [103].

Some efforts have been made in investigating the contribution of BPI polymorphisms in other diseases. While a genetic polymorphism in alpha 1-antitrypsin (A1AT) is linked to PR3-ANCA occurrence with a pathogenic role in systemic vasculitis [104], BPI polymorphisms do not appear to contribute to genetic predispositions for granulomatosis with polyangiitis disease [105]. Whether other BPI gene polymorphisms predispose to other diseases harboring chronic infections is still an area under investigation.

## 8 BPI therapeutic potential

Studies examining the possible therapeutic benefit of BPI often utilize recombinant N-terminal fragments of human BPI: rBPI_21_ or rBPI_23_. The only differences lie in the number of amino acids (rBPI_21_: 1–193, rBPI_23:_ 1–199), and the mutation of amino acid at position 132 (cysteine 132 is changed to alanine for rBPI_21_) to reduced dimer formation [106]. This mutation reduces rBPI_21_ heterogeneity and loss of activity is observed in rBPI_23_ while retaining the N-terminal bioactivities of BPI [107], leading to its entry into several clinical trials [[108], [109], [110], [111]]. Experimental animal models and preclinical and clinical studies in humans have demonstrated that exogenously administered recombinant BPI peptides intravenously can exert protective effects in the bloodstream [61,112,113]. Table 1 summarizes the role of BPI in different diseases.

**Table 1:** Bactericidal/permeability-increasing protein (BPI) and its role in different diseases. (References: CF: [19,20,39,75,76,88,101,102,124]; BE:^6,67^; COPD: [7,103,125]; Vasculitis: [63,[66], [67], [68],^79^,^105^,^126^,^127^]; IBD Crohn's: [77,78,82,[96], [97], [98]]; IBD UC: [77,78,94,[96], [97], [98]]; Sepsis/bacteremia: [84,89,95,114,122]; Pneumonia: [116,128]; Endotoxemia: [107]; Hemorrhage (trauma): [108]; Meningococcal disease: [12,[109], [110], [111],^120^].

Disease	Location of action	Function of BPI in these diseases	BPI autoantibody association	Prevalence of BPI autoantibody in selected patient cohorts	BPI gene polymorphism predisposition
Cystic fibrosis	Airways, lungs	Anti-inflammatory, anti-microbial, bacterial clearance	Yes	17.9–83% (49.45% pooled)	Yes
Bronchiectasis	Airways, lungs	Anti-inflammatory, anti-microbial, bacterial clearance	Yes	52–56%	Not reported
Chronic obstructive pulmonary disease	Airways, lungs	Anti-inflammatory, anti-microbial, bacterial clearance	Yes	48.15%	Yes
Vasculitis	Airways, kidneys	BPI-ANCA binding activates neutrophils, enhances vascular injury	Yes	45%	No
Inflammatory Bowel Disease: Crohn's	Intestinal tracts	Anti-inflammatory	Yes	14–75%	Yes
Inflammatory Bowel Disease: Ulcerative colitis	Intestinal tracts	Anti-inflammatory	Yes	29–75%	Yes
Sepsis/bacteremia	Systemic	Anti-inflammatory	Yes	46.7–64.7%	Yes
Pneumonia	Lungs	Anti-inflammatory, bacterial apoptosis	Not reported	17–38%	Not reported
Endotoxemia	Systemic	Anti-inflammatory	Not reported	Not reported	Not reported
Hemorrhage (trauma)	Site specific	Anti-inflammatory, anti-microbial	Not reported	Not reported	Not reported
Meningococcal disease	Systemic	Endotoxin clearance, bacterial inhibition	Not reported	Not reported	Not reported

### 8.1 Animal models

Intravenous administration of a recombinant rBPI_21_ in animal models of sepsis [114], pneumonia [115], and endotoxemia contributed to a significant reduction (>95% survival rate compared to <40% in control group) in mortality, associated with a reduction in serum LPS and TNF [114]. The combination of antibiotics with rBPI_21_ in an animal model of radiation-induced bone marrow aplasia was associated with survival rates of 65–80%, significantly greater than the 0–25% observed with control/antibiotics [113]. Intravenous infusion of rBPI_23_ has also been shown to reduce acute lung injury in endotoxemic pigs by ameliorating LPS-induced hypoxemia, functional upregulation of opsonin receptors on circulating phagocytes, and alveolitis [107]. Intraperitoneal injection of BPI has been shown to enhance *P. aeruginosa* uptake into the neutrophils, facilitates bacterial clearance from the peritoneal cavity, and reduce inflammation in mouse model deficient in BPI harboring acute peritoneal infection [129].

Promising data exist in other model systems. The therapeutic effects of BPI may not be limited to Gram-negative bacteria, as intranasal administration of rBPI_21_ in TLR-4 deficient mice infected with Gram-positive pathogen *Streptococcus pneumoniae*, led to enhanced upper respiratory tract bacterial apoptosis and prolonged survival [116].

Besides this possible therapeutic value in acute bacterial infections, BPI has shown utility in the treatment of burn wounds. Post-burn administration of rBPI_23_ reduced the incidence of bacterial translocation in mice [117]. Moreover, rBPI_23_ reduced neutrophil deposition in lungs and skin in rats after burn injury [118]. Additionally, BPI was shown to inhibit the infectivity of Influenza A virus strain H1N1, H3N2, and H5N1 due to its ability to modify the structure of virus particles leading to the breakdown of virus capsid, its ability to inhibit the replication of the virus, and its ability to inhibit the activation of human PBMCs by the virus shown in lower titers of IFNa and IL-6 [119]. These effects were seen only with human but not murine BPI [119]. This evidence further expands the scope of BPI therapeutic use.

### 8.2 Human studies

In patients with acute hemorrhagic trauma and meningococcal disease, clinical trials using rBPI_21_ have shown beneficial but limited effects of the recombinant N-terminus BPI fragment on the outcome of the disease [[108], [109], [110], [111]]. In a clinical trial study involving endotoxin challenge of human volunteers, rBPI_23_ neutralized endotoxin, suggesting rBPI_23_ is capable of attenuating the potentially deleterious effects of blood endotoxin in humans [61,112]. Moreover, rBPI_23_ also reduced the activation of the fibrinolytic and coagulation cascades after low-dose endotoxin infusion in human volunteers [113]. In children with severe meningococcal sepsis, rBPI_21_ was administered and proven to be effective in meningococci inhibition and bacterial endotoxin clearance, reducing clinically significant morbidities and improving the functional outcome of children with severe meningococcemia [110,120].

### 8.3 Therapeutic approaches

Due to rapid clearance from the circulation and short half-life of BPI *in vivo*, there are major limitations to the therapeutic utility of BPI and recombinant BPI fragments in the clinical settings [46,121]. This could possibly be the reason to why therapeutic usage of BPI did not go through late-stage clinical trials and into the market. Due to the functional nature of the amino terminus of BPI, rBPI_21_ and rBPI_23_ lacked the opsonic activity conferred by the C-terminus. Given our findings that human BPI in its full form containing both N- and C-terminus is essential for bacterial phagocytosis *in vivo* [129], with a limitation of short BPI half-life, other formulations of BPI that prolong its turnover time in circulation may tremendously benefit the functionality and practicality of BPI therapy. A chimeric protein consisting of N-terminal domain of lipopolysaccharide-binding protein (LBP) and the C-terminal domain of BPI demonstrated expanded duration of activity in circulation, as well as survival benefit and endotoxin reduction in neutropenic rats with *P. aeruginosa* sepsis [122]. Adeno-associated virus 2 (AAV2)-BPI_700_-fragment crystallizable gamma one 700 (Fcγ1_700_) chimeric gene transferred mice has shown a prolonged half-life of BPI *in vivo* and protection against minimal lethal dose of *E. coli* infection through BPI_1-199_-Fcγ1 protein expression [123]. This pharmacokinetic property of chimeric protein is beneficial for clinical dosing and administration. By increasing the time BPI can remain functionally active in circulation, this would allow broader utilization of the protein for therapeutic usage in patients.

## 9 Conclusion

Unlike antibiotic therapies that lack LPS neutralization properties and are prone to bacterial-resistance, BPI effects in killing bacteria, neutralizing bacterial endotoxins, while avoiding generation of antibiotic-resistant bacterial strains due to its membrane-targeting nature are proven to be useful for a new class of anti-bacterial therapy. With its nontoxic properties, bactericidal and opsonic activity, anti-inflammatory effects, and potential to exhibit synergistic interaction with conventional antibiotics, BPI remains a promising therapeutic molecule in mediating infection and inflammation in different diseases. Together with the fact that BPI is derived from the host itself, this makes it a safe and promising therapeutic molecule to be used against other Gram-negative bacterial infection in other diseases that may not be mentioned in this review. Clinical trials have indicated N-terminal domain of BPI to be effective for its bactericidal properties. There are still many more avenues to explore the C-terminal domain of BPI for its opsonophagocytosis properties. There is a possible risk for patients with anti-BPI antibodies for the BPI therapy to not be as effective, or that the introduction of BPI treatment could lead to anti-BPI antibodies generation. Presence of BPI autoantibodies and their linkage to worse disease outcome warrants further investigation into the mechanism leading to the induction of those antibodies as well as their roles in diseases.

## Credit author statement

Jomkuan Theprungsirikul: Conceptualization, Writing – original draft preparation. Sladjana Skopelja-Gardner: Writing- Reviewing and editing. William F. C. Rigby: Supervision, Funding acquisition, Writing- Reviewing and editing.

## Declaration of competing interest

The authors declare that they have no known competing financial interests or personal relationships that could have appeared to influence the work reported in this paper.
